# Influence of *Trichobilharzia regenti* (Digenea: Schistosomatidae) on the Defence Activity of *Radix lagotis* (Lymnaeidae) Haemocytes

**DOI:** 10.1371/journal.pone.0111696

**Published:** 2014-11-05

**Authors:** Vladimír Skála, Alena Černíková, Zuzana Jindrová, Martin Kašný, Martin Vostrý, Anthony J. Walker, Petr Horák

**Affiliations:** 1 Charles University in Prague, Faculty of Science, Department of Parasitology, Prague, Czech Republic; 2 Charles University in Prague, Faculty of Science, Institute of Applied Mathematics and Information Technologies, Prague, Czech Republic; 3 Charles University in Prague, 1st Faculty of Medicine, Institute of Immunology and Microbiology, Prague, Czech Republic; 4 Masaryk University, Faculty of Science, Department of Botany and Zoology, Brno, Czech Republic; 5 Institute of Haematology and Blood Transfusion, Prague, Czech Republic; 6 Molecular Parasitology Laboratory, School of Life Sciences, Kingston University, Kingston upon Thames, Surrey, United Kingdom; Natural Resources Canada, Canada

## Abstract

*Radix lagotis* is an intermediate snail host of the nasal bird schistosome *Trichobilharzia regenti*. Changes in defence responses in infected snails that might be related to host-parasite compatibility are not known. This study therefore aimed to characterize *R. lagotis* haemocyte defence mechanisms and determine the extent to which they are modulated by *T. regenti*. Histological observations of *R. lagotis* infected with *T. regenti* revealed that early phases of infection were accompanied by haemocyte accumulation around the developing larvae 2–36 h post exposure (p.e.) to the parasite. At later time points, 44–92 h p.e., no haemocytes were observed around *T. regenti*. Additionally, microtubular aggregates likely corresponding to phagocytosed ciliary plates of *T. regenti* miracidia were observed within haemocytes by use of transmission electron microscopy. When the infection was in the patent phase, haemocyte phagocytic activity and hydrogen peroxide production were significantly reduced in infected *R. lagotis* when compared to uninfected counterparts, whereas haemocyte abundance increased in infected snails. At a molecular level, protein kinase C (PKC) and extracellular-signal regulated kinase (ERK) were found to play an important role in regulating these defence reactions in *R. lagotis*. Moreover, haemocytes from snails with patent infection displayed lower PKC and ERK activity in cell adhesion assays when compared to those from uninfected snails, which may therefore be related to the reduced defence activities of these cells. These data provide the first integrated insight into the immunobiology of *R. lagotis* and demonstrate modulation of haemocyte-mediated responses in patent *T. regenti* infected snails. Given that immunomodulation occurs during patency, interference of snail-host defence by *T. regenti* might be important for the sustained production and/or release of infective cercariae.

## Introduction

Aquatic snails serve as intermediate hosts of many trematodes, including those important in veterinary and human medicine. Compatibility between such parasites and the host snail is partially governed by innate immunological processes that comprise cellular and humoral components. Mobile phagocytic cells called haemocytes play the major role in mediating the cellular defence response whereas lectins are considered as the most essential recognition molecules of humoral response [1], [2]. Haemocyte-mediated defence responses that are important for eliminating foreign invaders such as parasites include phagocytosis, encapsulation, and production of reactive oxygen species (ROS) [1], [3], [4].

Phagocytosis is used to eliminate small non-self particles, primarily bacteria; however, pieces of trematode tegument are also known to be actively engulfed by haemocytes after encapsulation [3]. The phagocytic response also triggers generation of ROS [5], [6]. Among the ROS, hydrogen peroxide (H_2_O_2_) is an important metabolite known for killing sporocysts of the human parasite *Schistosoma mansoni* [4]. At the molecular level, snail haemocyte defence responses are regulated by complex networks of intracellular signalling pathways, including the evolutionarily conserved protein kinase C (PKC) and mitogen-activated protein kinase (MAPK) pathways [7]–[10]. Activation of PKC, p38 MAPK and/or extracellular signal-regulated kinase (ERK) is required for efficient phagocytosis and H_2_O_2_ production by snail haemocytes; other kinases such as phosphatidylinositol 3-kinase also play a crucial role in these processes [7], [9]–[12].

During infection, compatible trematodes alter snail host defence responses presumably to help ensure survival and replication of the parasite. Phagocytic activity of haemocytes is decreased e.g. in the gastropods *Biomphalaria glabrata* and *Lymnaea stagnalis* infected with *Echinostoma paraensei* [13] and *Trichobilharzia szidati* [14], respectively. In the prosobranch snail, *Littorina littorea*, infection with *Himasthla elongata* reduces haemocyte ROS production, which correlates with increased haemocyte number in the snail circulation [15]. Such alterations of host defence mechanisms might be caused by trematode-derived components interfering with signalling pathways of snail haemocytes [16]. This hypothesis is supported by results showing that *S. mansoni* excretory-secretory products (ESPs) generated during development of miracidia to mother sporocysts impair H_2_O_2_ production in *B. glabrata* haemocytes [10] and disrupt ERK signalling in these cells [17].

*Radix lagotis* is an important intermediate host of the nasal bird schistosome *Trichobilharzia regenti* [18], [19], a causative agent of cercarial dermatitis in humans [20]. Following penetration into the snail, *T. regenti* miracidia develop to mother sporocysts, which in turn produce daughter sporocysts [21]. This latter stage gives rise to cercariae that are released into the water during the patent phase of infection. As far as immunological aspects of infection are concerned, snail defence responses related to the initiation of *T. regenti* infection, and changes in *R. lagotis* haemocyte activities in the patent phase of infection are unknown.

The present paper combines histological observations of juvenile *R. lagotis* snails infected with *T. regenti* miracidia, with comparisons of haemocyte abundance and haemocyte phagocytic activity and H_2_O_2_ production between uninfected and infected snails in the patent phase of *T. regenti* infection. At the molecular level, basal PKC and ERK phosphorylation in haemocytes from both snail groups was compared and their possible roles in regulation of haemocyte phagocytic activity and H_2_O_2_ production explored. Such complementary approaches provide the first and integrated insight into the immunobiology of *R. lagotis* snails demonstrating modulation of defence responses during infection of snails with the compatible trematode parasite.

## Methods

### Uninfected and *T. regenti*-infected *R. lagotis*

Uninfected *R. lagotis* were maintained in the laboratory at ambient room temperature (19–22°C; RT) in aquaria filled with aerated tap water and were fed fresh lettuce *ad libitum*. Juvenile and adult snails (together with the eggs laid) were reared together.

Juvenile snails with shell heights 5–8 mm were infected with *T. regenti* miracidia obtained as described by Horák *et al.* (1998) [18]. The snails were placed individually into wells of a 24-well culture plate (Nunc) containing tap water and each exposed to 3–8 miracidia for 5 h, with 15 miracidia used to infect each snail for histological analysis. After exposure, the snails were placed in a separate aquarium for 5 weeks, and they were then checked under a direct light source for shedding of *T. regenti* cercariae. Snails releasing cercariae (infected snails) were then maintained in a further separate aquarium.

### Light microscopy

Two juvenile *R. lagotis* were dissected for each infection time point studied, 1, 2, 3, 5, 12, 16, 20, 36, 44, 60 and 92 h post–exposure (p.e.) of snails to *T. regenti* miracidia. The soft body of each snail was carefully removed from its shell and fixed in Bouin-Hollande fixative at RT for 24 h. The specimens were then embedded in JB-4 resin (Polysciences), sections cut to 2 µm with a microtome (Finesse ME, Shandon Scientific) and stained with Wright-Giemsa (Polysciences). Finally, sections were individually embedded in DPX medium (Sigma), examined under an Olympus BX 51 light microscope and digital images captured using a DP70 digital camera system.

### Transmission electron microscopy

For transmission electron microscopy (TEM), juvenile *R. lagotis* were dissected 5 and 15 h p.e. and fixed in 2.5% glutaraldehyde (Sigma) in complete sterile snail saline (SSS+: 3 mM Hepes, 3.7 mM NaOH, 36 mM NaCl, 2 mM KCl, 2 mM MgCl_2_, 4 mM CaCl_2_, pH 7.8, 100 mOsm; [5]) at 4°C for 24 h. The specimens were then post–fixed in 1% OsO_4_ (Polysciences) in SSS+ for 2 h, washed three times in SSS+, dehydrated in ethanol (50%, 80%, 96%, twice each for 15 min, and 100% three times each for 5 min) and acetone (100%, three times each for 5 min). Subsequently, the tissue was incubated in 100% acetone∶Spurr mixture at increasing Spurr concentrations: 2∶1 for 2 h, 1∶1 for 5 h, 1∶2 for 12 h, followed by pure Spurr resin three times each for 12 h. Then, the material in fresh Spurr resin was transferred to plastic capsules and incubated at 60°C for 48 h. The embedded samples were first sectioned at 2 µm thick sections with a Finesse ME microtome, stained with 1% toluidine blue (Polysciences) and observed under a light microscope (Olympus BX 51). When larvae of *T. regenti* were detected, 60–70 nm thick sections were prepared using ultramicrotome Ultracut E (Reichert-Jung). These sections were stained with uranyl acetate and lead citrate [22] and evaluated under TEM JEOL 1011 microscope. Digital images were captured using associated software.

### Haemolymph extraction and enumeration of haemocytes in uninfected and infected *R. lagotis*

Uninfected and infected *R. lagotis* with shell heights 1.0–1.6 cm were selected for haemolymph extraction with infected snails extracted no later than 2 months post-patency. The snails were washed with distilled water, dried, and haemolymph was extracted by head-foot retraction [23].

Haemocyte numbers were quantified for individual uninfected and infected snails. Haemolymph from each snail was pooled on parafilm (Sigma) and diluted 1∶1, 2∶1, or 3∶1 (one part = 10 µl) in incomplete sterile snail saline (SSS-) where 2 mM MgCl_2_ and 4 mM CaCl_2_ were omitted, and 2% ethylenediaminetetra-acetic acid (EDTA; Sigma) added (SSS-/EDTA) to reduce haemocyte aggregation/adhesion; SSS-/EDTA buffer was exclusively used for counting haemocytes. Enumeration was carried out with Bürker haemocytometers and haemocyte numbers were expressed as haemocytes/ml of haemolymph. The data were analysed for normality (Shapiro-Wilk normality test) using R 2.13.0 statistical software (www.r-project.org). Spearman's correlation test was used to assess the relationship between shell heights and haemocyte numbers of individual snails. Haemocyte numbers between the snail groups were compared using Wilcoxon signed-rank test (non-parametric two-sample test; Wilcoxon test).

### Preparation of haemocyte monolayers

Haemolymph from uninfected and infected snails (shell heights 1.3–1.6 cm) was extracted in alternating order to ensure similar conditions for both haemolymph types while the monolayers were prepared. Aliquots of haemolymph drawn from the snails were pipetted directly into the wells of a 96-well culture plate (Nunc) containing 50 µl SSS+ to achieve a final volume of 250 µl/well (final ratio: 4 parts haemolymph: 1 part SSS+). Ten to forty snails were required to obtain sufficient haemolymph for each monolayer. Haemocytes were left to settle and adhere to the bottom of the wells for 30 min at RT. Monolayers were then washed with SSS+ (see below) and their quality checked under a microscope (Olympus IX 71). Any wells containing haemocyte clumps or discontinuous monolayers were not used. When haemocyte numbers per well were enumerated, aliquots of haemolymph were also collected on parafilm and diluted with equal amount of SSS-/EDTA; haemocytes were then enumerated as described above.

### Phagocytosis assays

Haemocyte monolayers were washed three times with 250 µl SSS+ and equilibrated in 190 µl SSS+ for 30 min at RT. 10 µl of *Escherichia coli* bioparticles (pHrodo red; Molecular Probes) prepared following manufacturer's instructions were then added to each well and plates incubated at RT in the dark for 2 h. These bioparticles are non-fluorescent outside cells, but become fluorescent in phagosomes. Therefore, no washing was necessary after incubation and intracellular fluorescence was immediately quantified using Tecan Infinite M200 microplate reader at 545 nm excitation and 600 nm emission. The signal of *E. coli* bioparticles alone in wells was also measured in each assay and the value subtracted from all values obtained from wells containing haemocytes and *E. coli* bioparticles.

Phagocytic activity of haemocytes from uninfected and infected snails was then expressed per volume of haemolymph (200 µl) and per 50,000 haemocytes, in case infection altered haemocyte number. Uninfected snails were also used to study the effects of inhibition of PKC and ERK signalling on phagocytic activity. Haemocyte monolayers were pre-incubated for 30 min at RT with 1 µM or 10 µM inhibitor of PKC (GF109203X; Sigma), MEK (U0126; Cell Signalling Technology - CST), which is the immediate upstream activator of ERK, or in DMSO vehicle alone (0.05%; Sigma) prior to adding bioparticles. Effects of inhibition assays were evaluated in terms of haemolymph volume (200 µl).

Using R 2.13.0 statistical software, raw fluorescence intensity data for each measurement were analysed for normality (Shapiro-Wilk normality test). Wilcoxon test was then used to compare the phagocytic activity between uninfected and infected snails, whereas paired t-test was applied to data when assessing the effect of GF109203X and U0126 on phagocytosis by *R. lagotis* haemocytes. For graphic representation, the data for uninfected snails were assigned a value of 100%.

### Hydrogen peroxide assays

Haemocyte monolayers were prepared and haemocyte numbers/well enumerated as described above except that 50 µl haemolymph and 12.5 µl SSS+ were used per well. After washing monolayers twice with 250 µl SSS+, haemocytes were left to equilibrate for 30 min at RT in 100 µl SSS+. H_2_O_2_ output by haemocytes was monitored using the Amplex red hydrogen peroxide/peroxidase assay kit (Molecular Probes) in which Amplex red reacts with H_2_O_2_ to produce the red-fluorescent product, resorufin. Working solutions of the assay mixture that were prepared in SSS+ contained: 0.1 U ml^−1^ horseradish peroxidase (HRP), 50 µM Amplex red reagent, and either 0.1% DMSO or 10 µM PMA (phorbol 12-myristate 13-acetate; Sigma) in DMSO. PMA was used because in other molluscs this phorbol ester increases ROS production by haemocytes [10], [24], [25]. 100 µl of the respective working solution was added to each individual haemocyte monolayer and the plate was incubated in the dark for 30 min at RT. For inhibition assays using uninfected snails, haemocytes were exposed to 5 µM GF109203X, U0126 or DMSO (vehicle) alone (0.025%) for 30 min at RT prior to adding the working solution containing PMA. The final concentration of DMSO after adding the working solutions was 0.1% in all cases.

Fluorescence was monitored at 520 nm and 615 nm excitation and emission, respectively, in a microplate reader (Tecan Infinite M200) for 60 min. H_2_O_2_ output by uninfected and infected snail haemocytes was evaluated per volume of haemolymph (50 µl) and haemocyte number with adjustment to 50,000 cells. Inhibition assays were evaluated per volume of haemolymph (200 µl).

The data sets were tested for normality (Shapiro-Wilk normality test) and for equality of variances (Two-variances F-test). Two-sample t-test or Wilcoxon test was used to compare basal and PMA-modulated H_2_O_2_ production between uninfected and infected snails. Experiments investigating the effects of PKC and ERK inhibition on H_2_O_2_ production were analysed using either parametric or nonparametric paired tests. Since the tests at different time points are dependent, a Fisher's combination test using inverse normal method [26] was used for further processing of p-values. The resulting test statistic was compared to Pocock's critical value 2.49. If the test statistic was higher than this critical value, a significant difference between data sets was confirmed.

### SDS-PAGE and western blot analysis

Haemocyte monolayers prepared as detailed above were washed three times with 250 µl SSS+, and left to equilibrate in 250 µl SSS+ at RT for 30 min. The SSS+ was then removed and haemocytes lysed by adding 25 µl of hot (95°C) SDS-PAGE sample buffer. Proteins were separated by gel electrophoresis (10% Mini-Protean TGX precast gel; Bio-Rad) and transffered to Immun-Blot PVDF membrane (Bio-Rad) using Trans-Blot turbo blotting system (Bio-Rad). Membranes were blocked with 5% non-fat dried milk (Bio-Rad) in 0.1% Tween/Tris-buffered saline (TTBS) at RT for 45 min, and incubated overnight at 4°C in either anti-phospho-PKC (pan) (βII Ser660) rabbit polyclonal antibodies or anti-phospho-p44/42 MAPK (Erk1/2) (Thr202/Tyr204) (197G2) rabbit monoclonal antibodies (CST) (1∶1000 in TTBS). These antibodies were previously validated for detection of exclusively phosphorylated (activated) forms of PKC and ERK in *L. stagnalis* haemocytes [7], [27], and were also used in other studies of molluscs [17]. Following further incubation at RT for 2 h, membranes were washed 3×5 min in TTBS and incubated for 2 h at RT in anti-rabbit IgG HRP-conjugated secondary antibodies (1∶4000 in TTBS) (CST). Immunoreactive bands were then visualised using SuperSignal West Dura extended duration substrate (Thermo Scientific) and a LAS 4000 Luminescent image analyser. Blots were stripped in Restore Western blot stripping buffer (Thermo Scientific) for 2 h at RT, and re-probed overnight in p44/p42 MAPK (Erk1/2) antibody (CST) (1∶1000 in TTBS), which recognizes ERK regardless of its phosphorylation state. Finally, the blots were stripped and re-probed with anti-actin antibodies (Sigma) (1∶4000 in TTBS) for 1 h at RT to confirm equal loading of proteins between lanes.

The intensities of immunoreactive bands were analysed using Multi Gauge 3.2. software. The values for PKC and ERK phosphorylation and for total ERK in haemocytes of uninfected snails were standardised as 100% and differences in PKC and ERK phosphorylation and in total ERK from infected snails calculated. The data were evaluated for normality (Shapiro-Wilk normality test) and for equality of variances (Two-variances F-test). Two-sample t-test was then applied using R 2.13.0 statistical software.

## Results

### Histological observations of *R. lagotis* experimentally infected with *T. regenti*

**Figure 1:**
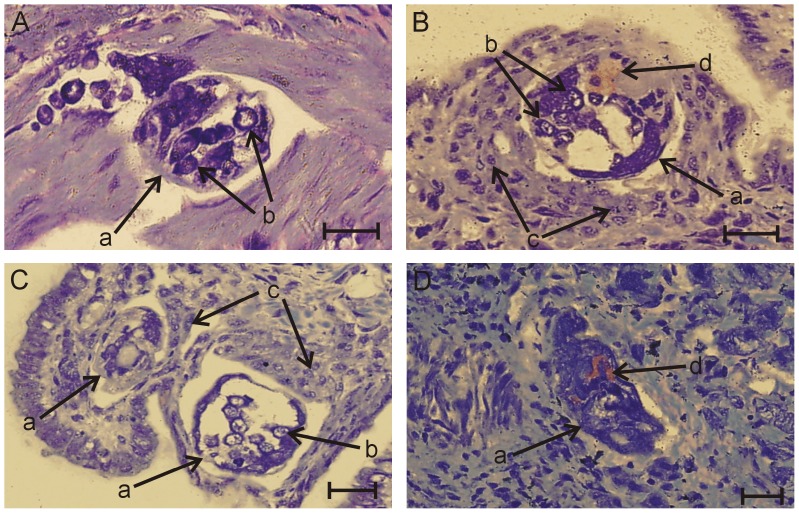
*Trichobilharzia regenti* larvae within the tissue of *Radix lagotis* revealed by light microscopy between 1–92 h p.e.; Wright-Giemsa stained sections. (A) Miracidium of *T. regenti* (a) containing germ cells (b) occurs within the snail tissue without haemocyte infiltration 1 h p.e. (B) and (C) Haemocytes (c) are present in the vicinity of developing *T. regenti* mother sporocyst (a) 2 and 16 h p.e., respectively; germ cells (b) and gland structure (d) of the parasite are visible. (D) The area around *T. regenti* mother sporocyst (a) contains no haemocytes 92 h p.e. Gland structure (d) is located in the body of the parasite. Scale bar = 20 µm. The images shown are representative of the situation seen in all sections observed during these experiments.

Histological observations of *R. lagotis* experimentally infected with *T. regenti* provided insights into the encapsulation responses within the snail tissue between 1 and 92 h p.e. Haemocytes were not evident in close proximity to the parasite at 1 h p.e. (Figure 1A). However, considerable accumulation of haemocytes was observed close to the developing *T. regenti* between 2 and 16 h p.e. (Figure 1B–C). Haemocytes appeared to surround the developing mother sporocysts irregularly in several layers; however, it was not clear whether the cells were directly attached to the parasite surface. Thereafter, at 20 and 36 h p.e. the haemocytic response against the parasite appeared to decline (data not shown) and while the haemocytes occurred individually in the vicinity of mother sporocysts, they did not accumulate in layers. At the latter time points, 44, 60 and 92 h p.e. no haemocytes were observed close to *T. regenti* (Figure 1D).

**Figure 2:**
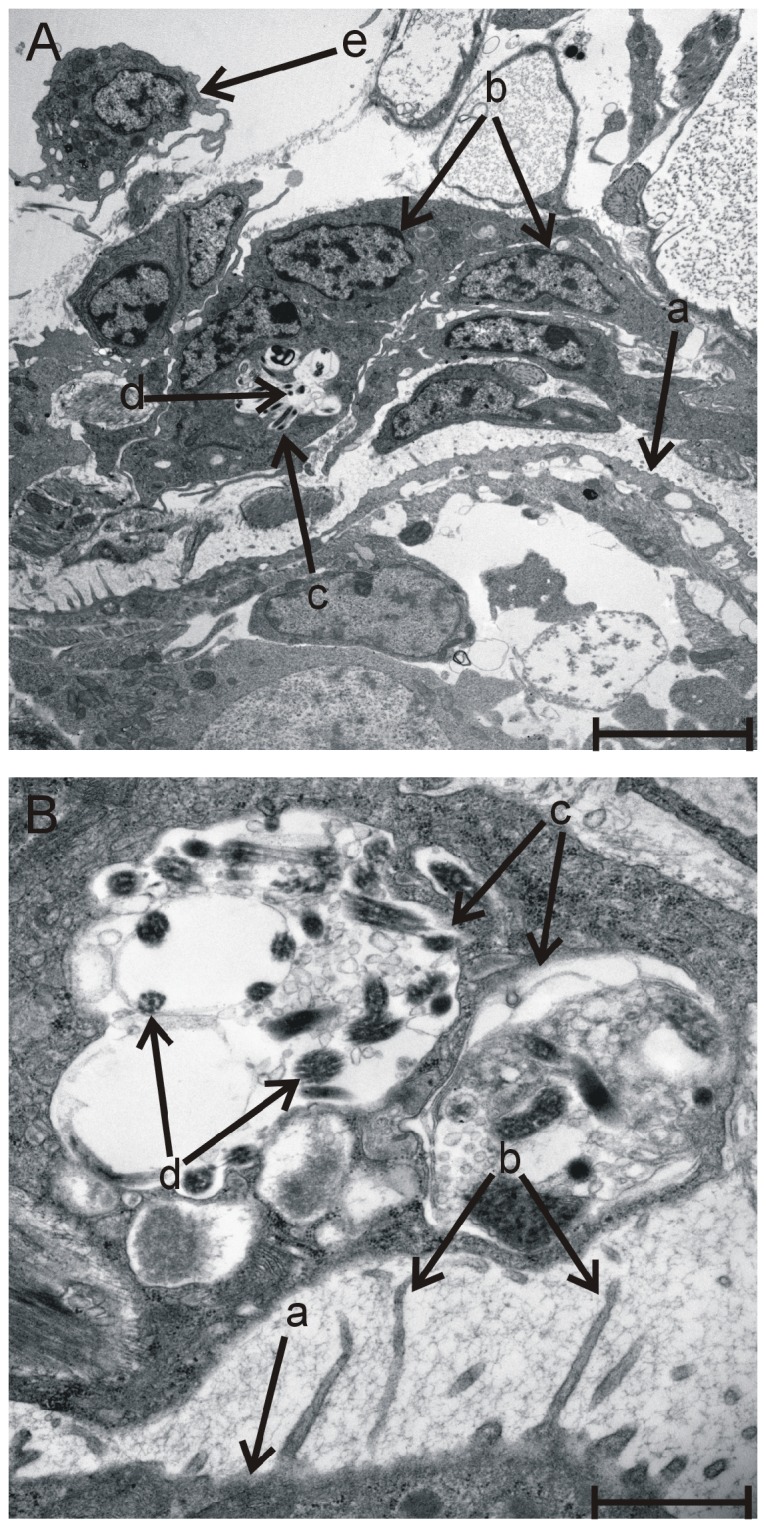
*Trichobilharzia regenti* mother sporocysts within the tissue of *Radix lagotis* 15 h p.e.; TEM images. (A) Mother sporocyst of *T. regenti* (a) is surrounded by haemocytes with remarkable nuclei (b). Phagosome (c) of one haemocyte with internalised microtubular aggregates (d) is visible (B in detail). Another haemocyte (e) is located near the parasite. Scale bar = 5 µm. (B) Microvilli (b) are present on the surface of *T. regenti* mother sporocyst (a). Haemocyte adjacent to the sporocyst contains phagosomes (c) with microtubular aggregates (d). Scale bar = 1 µm.

Transmission electron microscopy of *T. regenti* mother sporocysts within the snail tissue at 5 and 15 h p.e. (Figure 2; 15 h p.e. shown) showed that the larvae remained apparently undamaged despite numerous haemocytes being adjacent to the parasite (Figure 2A). Furthermore, some haemocytes were in a tight contact with sporocyst surface microvilli, and microtubular aggregates were observed within their phagosomes (Figure 2A–B).

### Haemocyte number in uninfected and *T. regenti*-infected *R. lagotis*

**Figure 3:**
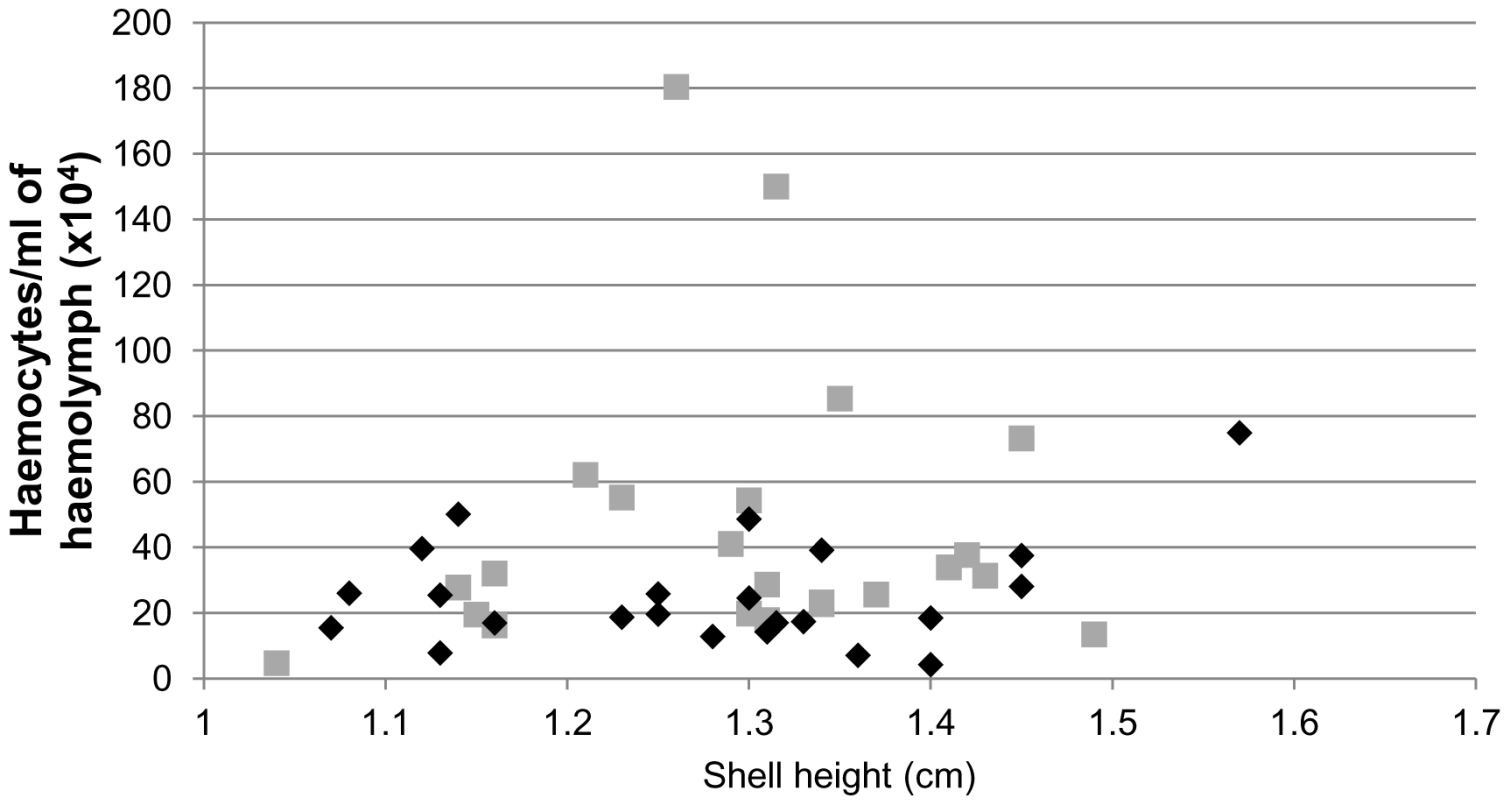
Number of haemocytes/ml of haemolymph of individual uninfected (black diamond) and *Trichobilharzia regenti* infected (grey box) *Radix lagotis*. The numbers of haemocytes/ml from individual snails with different shell heights were enumerated using a Bürker haemocytometer.

Evaluation of haemocyte number/ml haemolymph in 23 individuals of uninfected and infected *R. lagotis* demonstrated that the concentration of circulating haemocytes did not correlate with the shell height of the snails (Figure 3). Considerable variation in haemocyte number was observed within the extracted haemolymph for snails of similar size. In uninfected snails, the lowest haemocyte concentration was 4.2×10^4^ cells/ml (shell height 1.40 cm) whereas the highest was 74.9×10^4^ cells/ml (shell height 1.57 cm) (Figure 3). In infected snails, the lowest haemocyte concentration was 4.7×10^4^ cells/ml (shell height 1.04 cm) whereas the highest was 180.4×10^4^ cells/ml (shell height 1.26 cm) (Figure 3). Statistical analysis revealed that mean haemocyte number/ml haemolymph of infected snails was 79% greater than that of uninfected snails (45.9×10^4^ cells/ml vs. 25.6×10^4^ cells/ml; p<0.05).

### Defence responses of haemocytes from uninfected and *T. regenti*-infected *R. lagotis*

**Figure 4:**
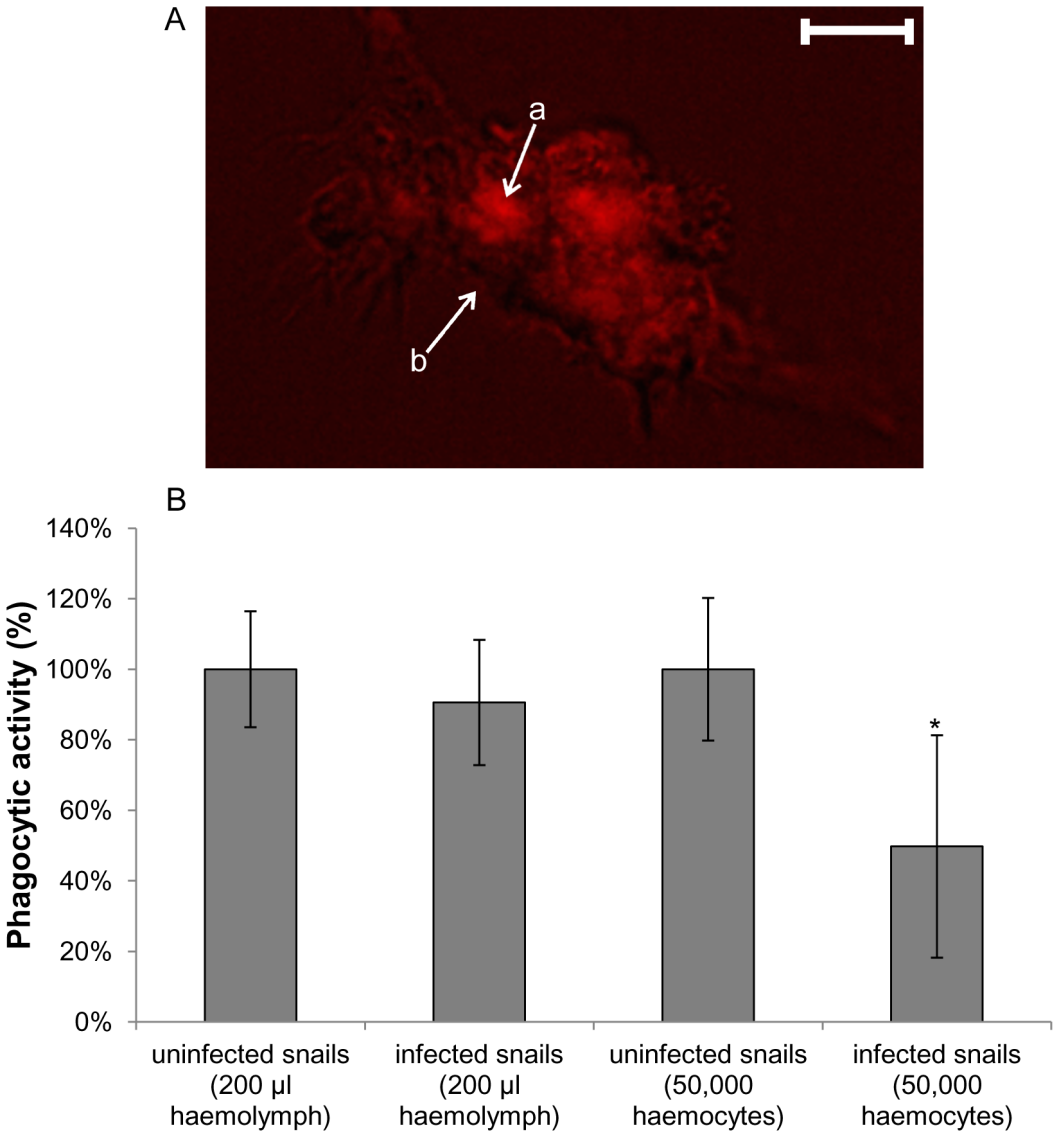
Phagocytosis of *E. coli* bioparticles by haemocytes from uninfected and *Trichobilharzia regenti* infected *Radix lagotis*. Phagocytic activities were assessed by incubating *E. coli* bioparticles with haemocyte monolayers and assessing the relative fluorescence of internalised particles after 2 h using a microplate reader. (A) The combined (phase-contrast and fluorescence) image of *E. coli* bioparticles (a) within a haemocyte (b); scale bar = 10 µm. (B) Data were evaluated per volume of haemolymph (200 µl) and per number of haemocytes (50,000) (shown as mean values ± SEM; n = 7) with uninfected snails considered as having 100% activity. *p<0.05 when compared to uninfected snails (50,000 haemocytes); Wilcoxon test.

To explore the effects of *T. regenti* infection on haemocyte defence, we measured phagocytic activity and H_2_O_2_ production by haemocytes derived from uninfected and *T. regenti*-infected *R. lagotis*. Haemocyte phagocytic activity was determined by the ability of these cells to internalise *E. coli* bioparticles (Figure 4A). Comparisons made in a physiological context, which consider activity per volume of haemolymph (200 µl), revealed that phagocytosis by haemocytes from infected snails was not significantly different from that of uninfected snails (Figure 4B). However, when the phagocytic activity was compared taking into account the different numbers of haemocytes in the extracted haemolymph, with more haemocytes present as a result of parasite infection, phagocytosis by infected snail haemocytes was reduced significantly to approximately 50% of that of uninfected snails (p<0.05; Figure 4B).

**Figure 5:**
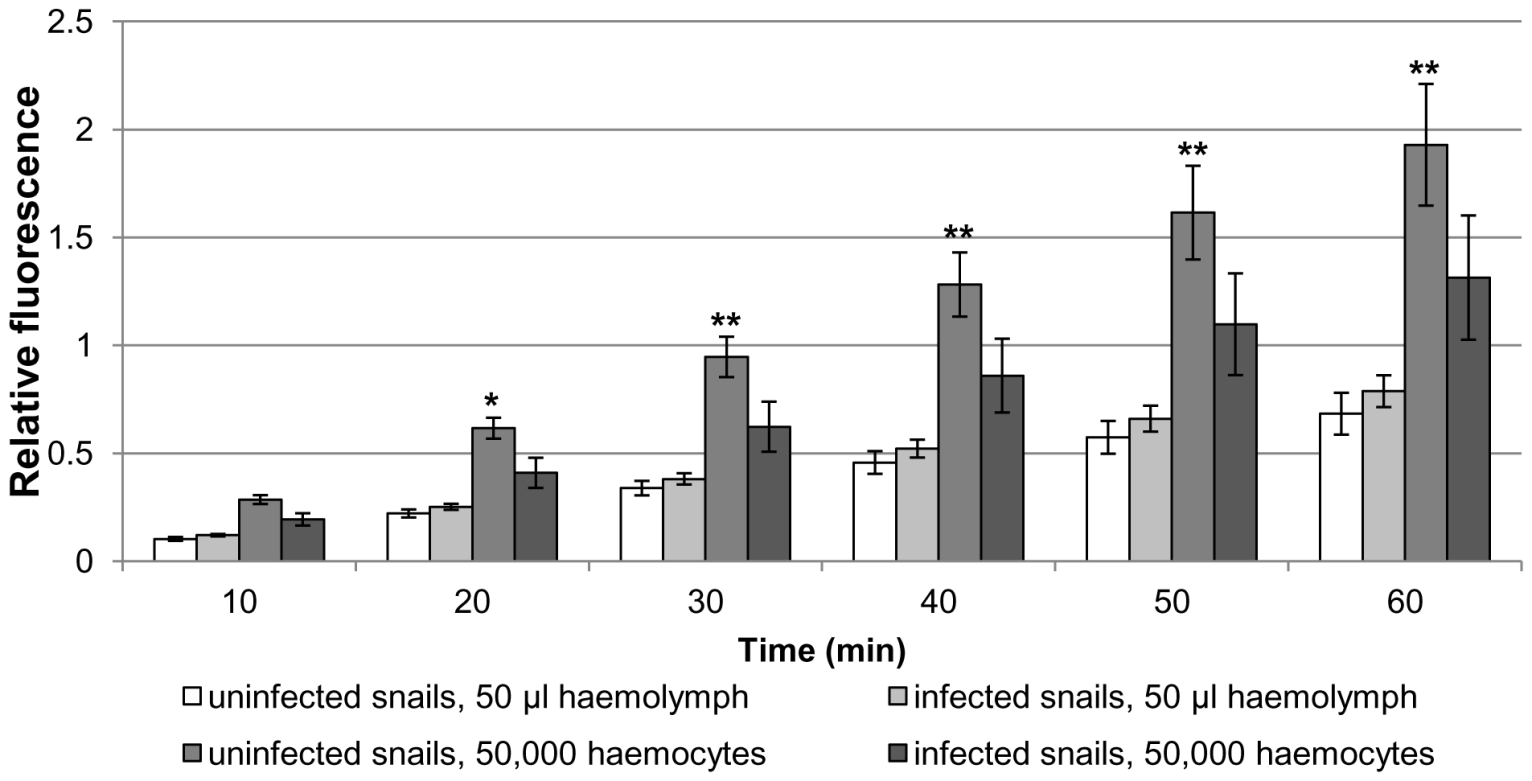
Basal H_2_O_2_ production in haemocytes from uninfected and *Trichobilharzia regenti* infected *Radix lagotis*. H_2_O_2_ output by haemocyte monolayers was detected by Amplex red and the intensity of fluorescence measured by microplate reader over 60 min. The mean relative fluorescence values are shown (± SEM; n = 7) and represent the increase in H_2_O_2_ production over time. Data were evaluated per volume of haemolymph (50 µl) and per number of haemocytes (50,000). *p<0.05, **p<0.01, when compared to infected snails (50,000 haemocytes); two-sample t-test or Wilcoxon test combined with Fishers's combination test.

**Figure 6:**
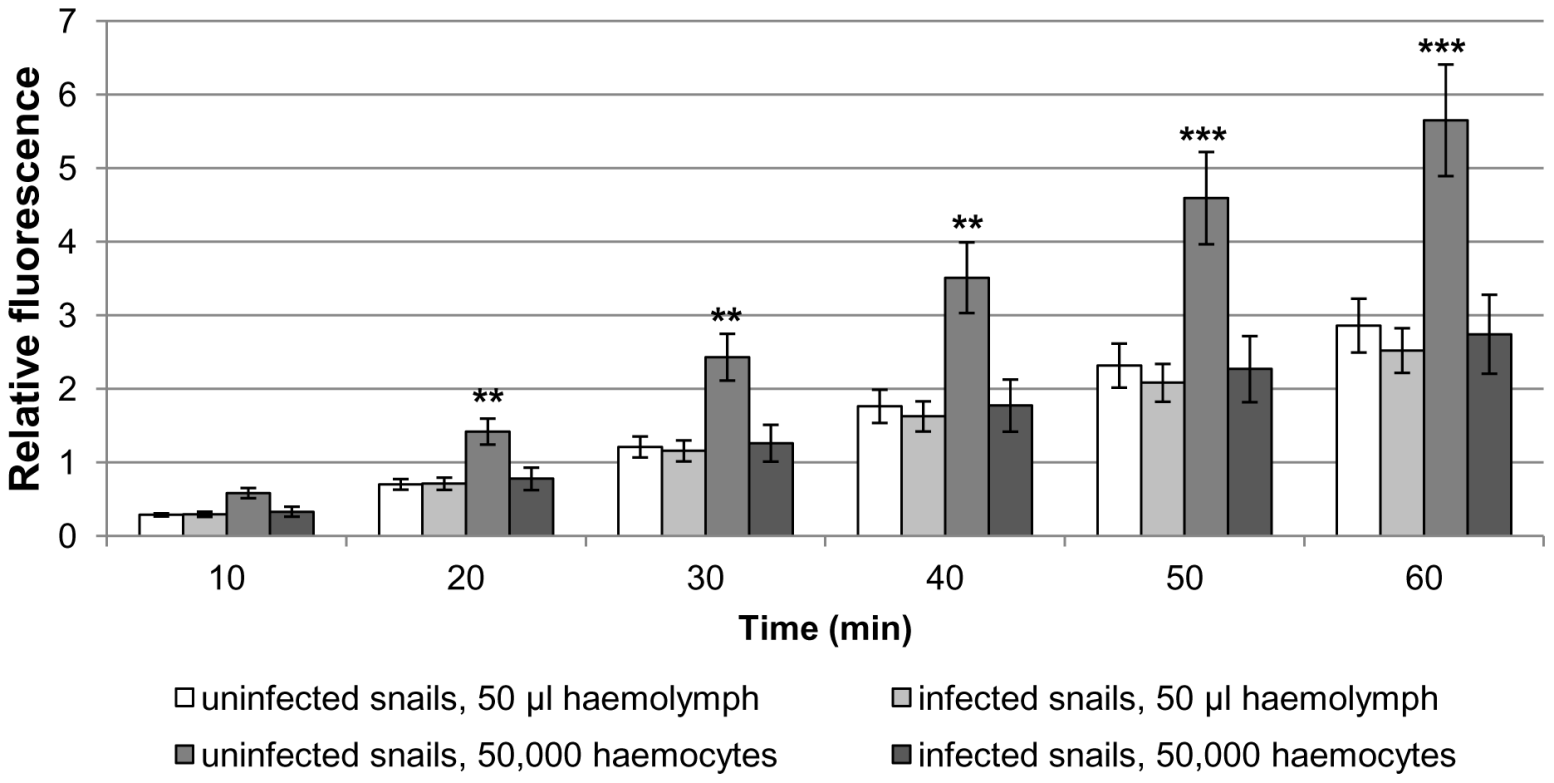
PMA-stimulated H_2_O_2_ production in haemocytes from uninfected and *Trichobilharzia regenti* infected *Radix lagotis*. H_2_O_2_ output by haemocyte monolayers treated with 5 µM PMA was detected by Amplex red and the intensity of fluorescence was measured by microplate reader over 60 min. The mean relative fluorescence values are shown (± SEM; n = 7) and represent the increase in H_2_O_2_ production over time. Data were evaluated per volume of haemolymph (50 µl) and per number of haemocytes (50,000). **p<0.01, ***p<0.001, when compared to infected snails (50,000 haemocytes); two-sample t-test or Wilcoxon test combined with Fishers's combination test.

For H_2_O_2_ production we studied basal and PMA-stimulated output by haemocytes from uninfected and infected snails (Figures 5–6). Evaluation per volume of haemolymph (50 µl) revealed that the basal output of H_2_O_2_ by haemocytes from infected snails was similar to that of uninfected snails, despite the infected snails possessing greater numbers of haemocytes/ml (Figure 5). In contrast, when the data were adjusted for haemocyte number (50,000), the cells from uninfected snails produced significantly more H_2_O_2_ than those from infected snails at each time point after 20 min (p<0.05; Figure 5).

In the presence of 5 µM PMA (an activator of PKC) haemocyte H_2_O_2_ production increased 270% and 240% when considering haemolymph volume (50 µl) in uninfected and infected snails after 60 min, respectively (Figure 6); the difference between snail groups was not statistically significant. In contrast, when considering haemocyte number (50,000) H_2_O_2_ production by haemocytes from uninfected snails in the presence of PMA was approximately 2-fold that of haemocytes from infected snails at all time points studied after 20 min (p<0.01; Figure 6).

### PKC and ERK activation in haemocytes from uninfected and *T. regenti*-infected *R. lagotis*

**Figure 7:**
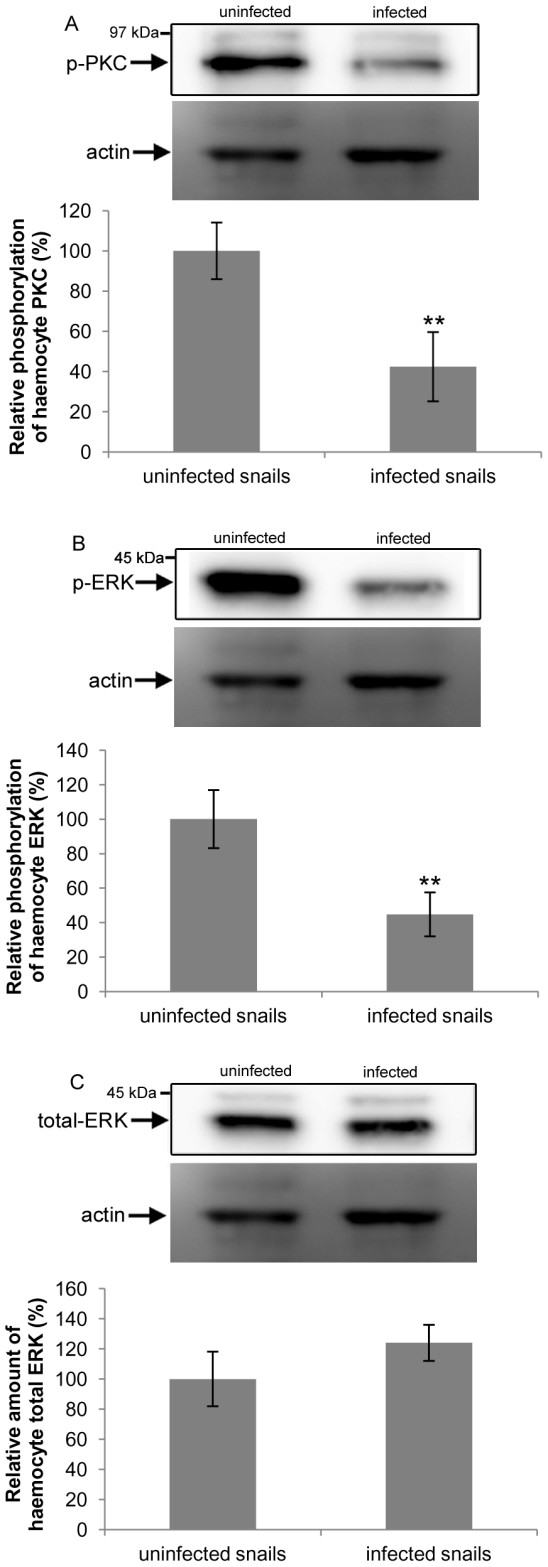
PKC and ERK phosphorylation and total ERK levels in haemocytes from uninfected and *Trichobilharzia regenti* infected *Radix lagotis*. Representative blots showing (A) PKC and (B) ERK phosphorylation in adherent haemocytes from uninfected and infected snails. (C) Levels of total ERK in uninfected and infected snails. Band intensities were measured and the mean (± SEM) haemocyte PKC and ERK phosphorylation (n = 10) and total ERK levels (n = 5) calculated (shown in the graphs) with uninfected values considered as 100%. **p<0.01 when compared to haemocyte PKC and ERK phosphorylation levels in uninfected snails; two-sample t-test.

Because signalling pathways are known to regulate haemocyte defence responses such as phagocytosis and H_2_O_2_ output [7], [9]–[12], and because these defence responses were supressed in *R. lagotis* haemocytes as a result of *T. regenti* infection, we aimed to determine PKC and ERK activation in haemocyte monolayers derived from uninfected and infected *R. lagotis*. Western blotting of haemocyte proteins with anti-phosphospecific PKC and ERK antibodies, which detect only the active forms of these kinases in snails [7], [8], [27], followed by densitometric analysis of immunoreactive bands from several independent blots revealed that PKC and ERK phosphorylation were reduced by 57% and 55%, respectively, in haemocytes from infected snails when compared to those from uninfected snails (p<0.01; Figure 7A–B). We reasoned, therefore that ERK expression might also be suppressed. However, western blots performed to determine the quantity of ERK in haemocytes using antibodies that detect ERK irrespective of its phosphorylation state (Figure 7C) demonstrated that mean levels of ERK were 24% higher in infected snails when compared to uninfected ones, although this difference was not statistically significant. Unfortunately, lack of a suitable anti-PKC antibody for snails prevented evaluation of total PKC protein levels.

### Effect of PKC and MEK inhibitors on phagocytosis and H_2_O_2_ production

**Figure 8:**
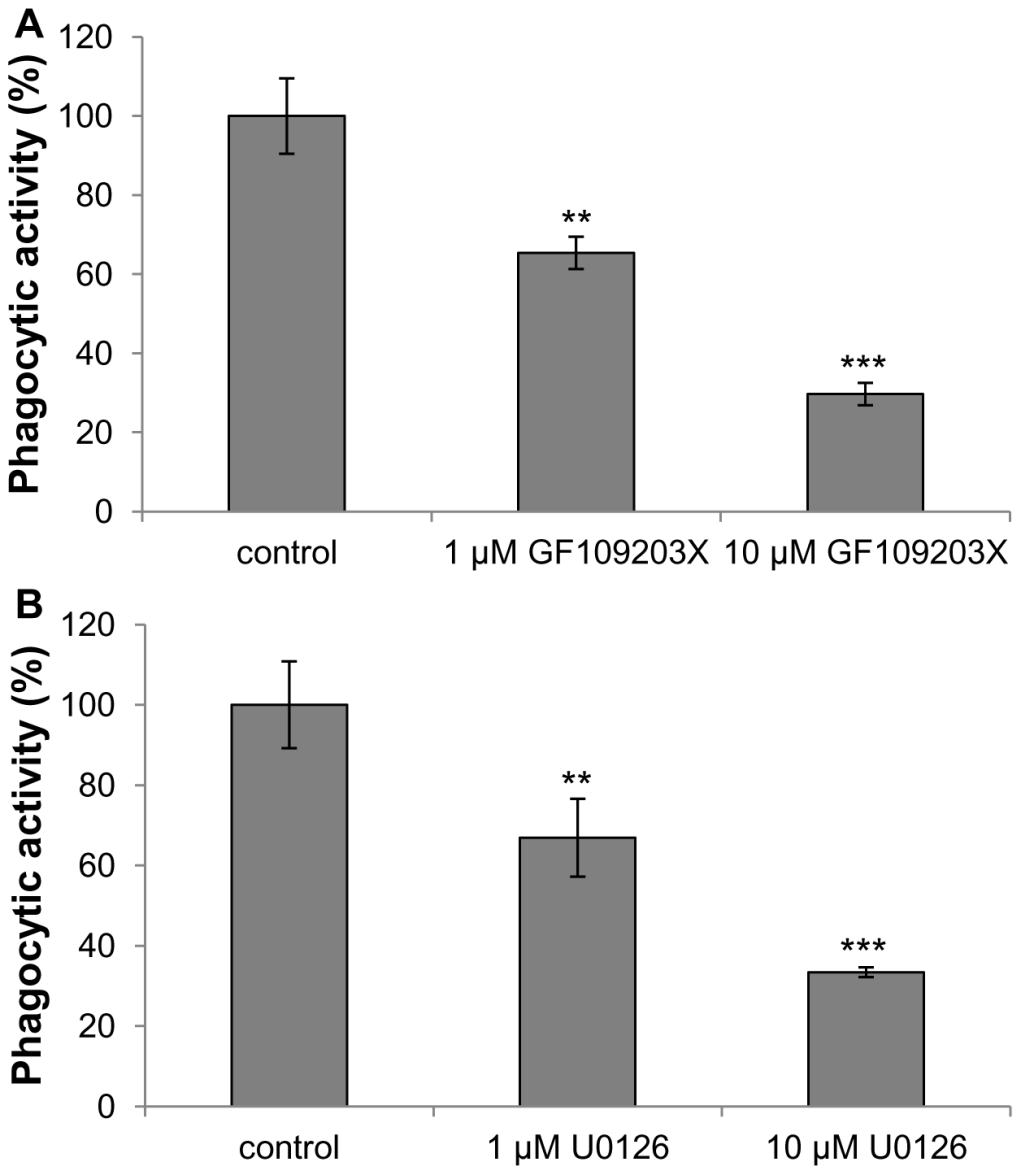
Effect of PKC (GF109203X) and MEK (U0126) inhibitors on phagocytosis by haemocytes from uninfected *Radix lagotis*. Haemocyte monolayers were pre-incubated with (A) GF109203X, (B) U0126, or vehicle (DMSO; shown as controls) prior to challenge with *E. coli* bioparticles. The intracellular fluorescence resulting from phagocytosis was measured using a microplate reader and mean (± SEM; n = 7) levels of phagocytosis expressed in relation to control (100%) values. **p<0.01, ***p<0.001, when compared to control values; paired t-test.

To investigate the possible role of PKC and ERK in the regulation of phagocytosis by *R. lagotis* haemocytes, cells from uninfected snails were incubated with the PKC or MEK inhibitors GF109203X or U0126, respectively, compounds that have been shown to decrease PKC or ERK phosphorylation (activation) in *L. stagnalis* haemocytes [7], [27]. Phagocytosis was blocked in a dose-dependent manner, with 1 µM and 10 µM GF109203X significantly suppressing uptake of bioparticles by approximately 35% and 70%, respectively (p<0.01, p<0.001; Figure 8A). U0126 at 1 µM and 10 µM concentration significantly reduced phagocytic activity of haemocytes by approximately 33% and 67%, respectively (p<0.01, p<0.001; Figure 8B).

**Figure 9:**
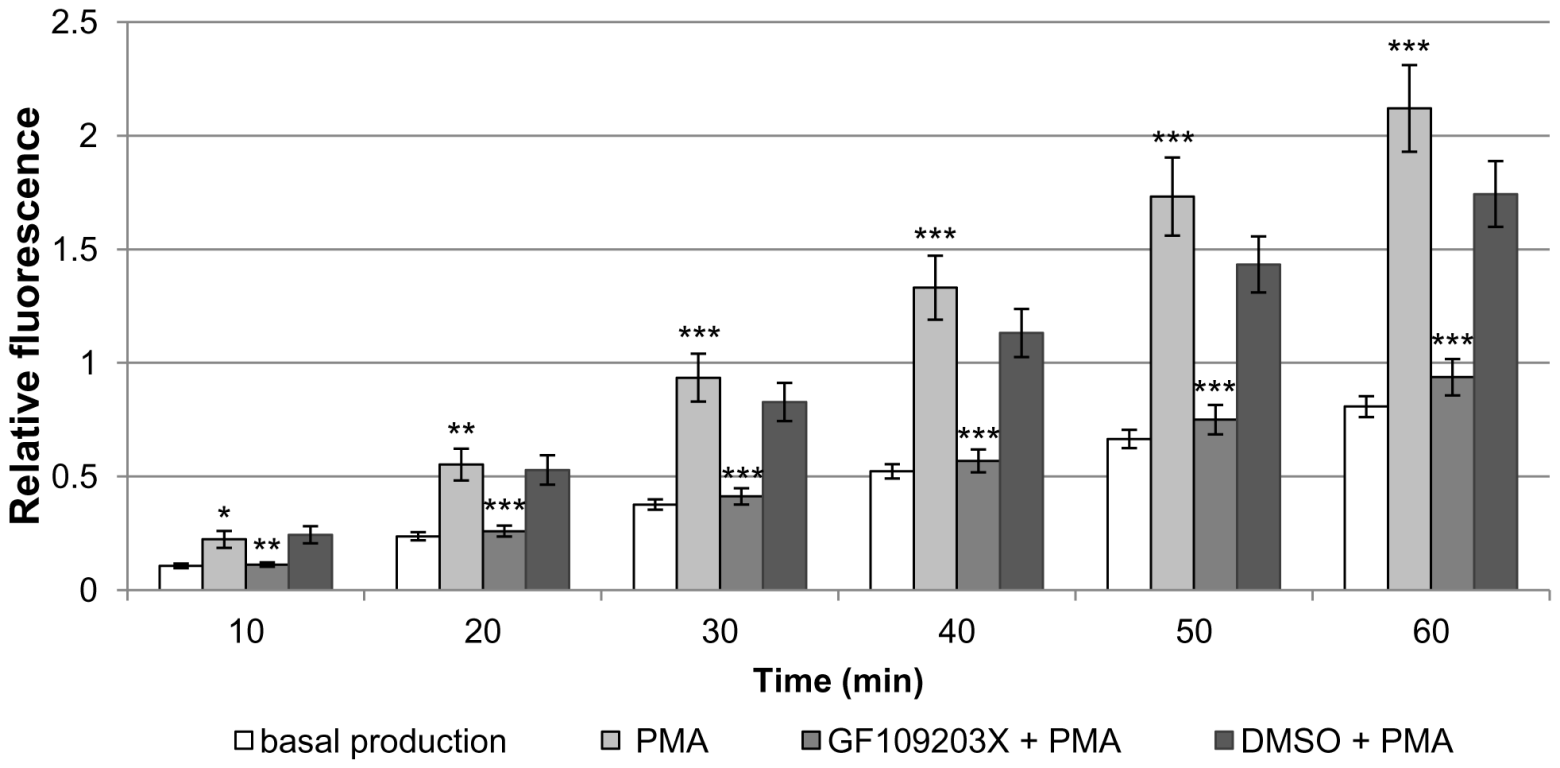
PMA-stimulated H_2_O_2_ production in haemocytes from uninfected *Radix lagotis*, and the effect of PKC inhibition on H_2_O_2_ production. H_2_O_2_ output by haemocyte monolayers in the presence of PMA (5 µM), GF109203X (5 µM) and PMA, DMSO (vehicle) and PMA, or SSS+ alone was detected by Amplex red and the intensity of fluorescence was measured by microplate reader over 60 min. The mean (± SEM; n = 7) relative fluorescence values shown represent the increase in H_2_O_2_ production over time in the various treatments. *p<0.05, **p<0.01, ***p<0.001, for PMA values compared to basal production, and **p<0.01, ***p<0.001 for GF109203X+PMA compared to DMSO+PMA; paired t-test or paired-samples Wilcoxon test combined with Fishers's combination test.

**Figure 10:**
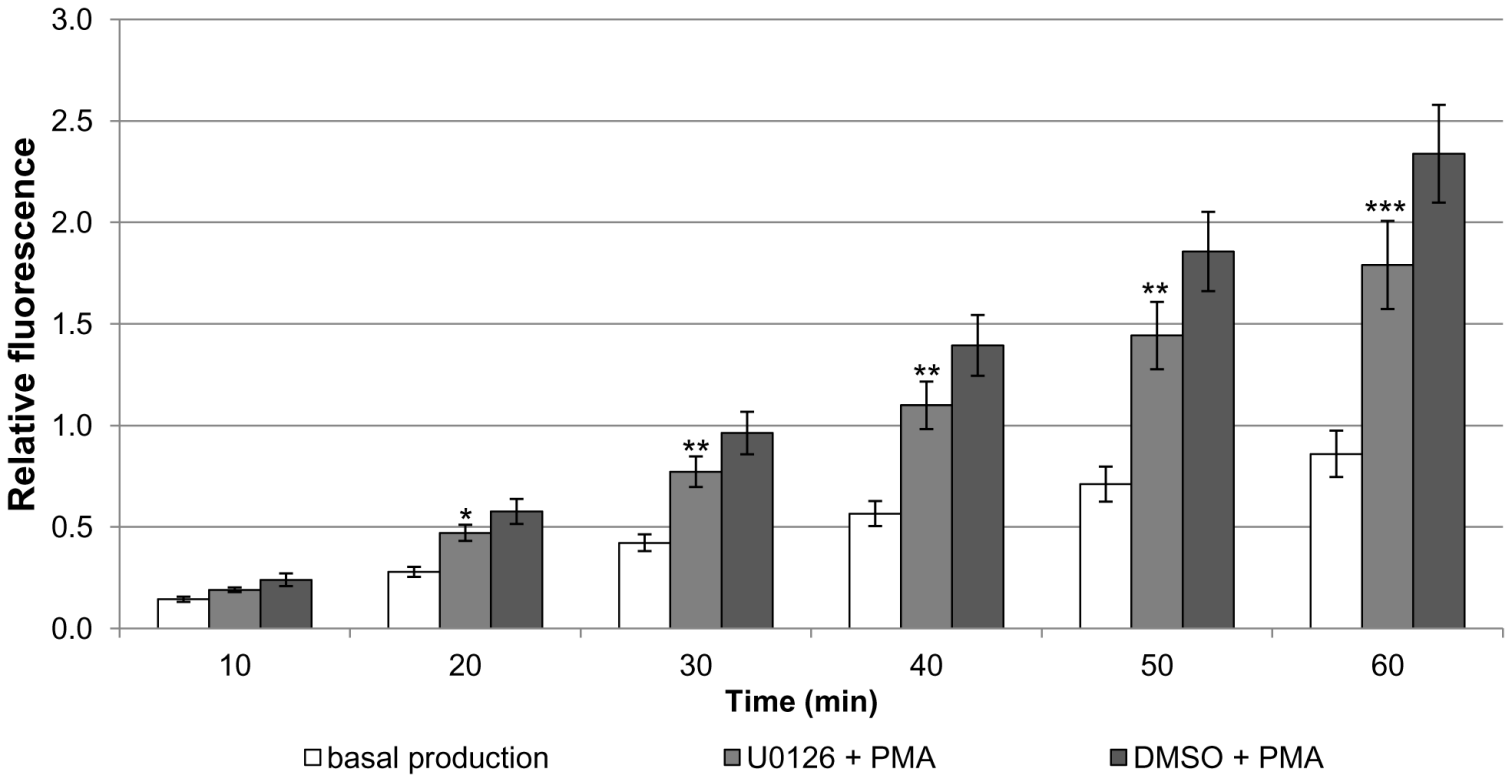
The effect of MEK inhibition on PMA-stimulated H_2_O_2_ production in haemocytes from uninfected *Radix lagotis*. H_2_O_2_ output by haemocyte monolayers in SSS+ alone, U0126 (5 µM) and PMA, or DMSO (vehicle) and PMA was detected by Amplex red and the intensity of fluorescence was measured by microplate reader over 60 min. The mean (± SEM; n = 3 for SSS+ otherwise n = 7) relative fluorescence values shown represent the increase in H_2_O_2_ production over time in the various treatments. *p<0.05, **p<0.01 and ***p<0.001 for U0126+PMA compared to DMSO+PMA; paired t-test or paired-samples Wilcoxon test combined with Fishers's combination test.

Treatment of haemocytes from uninfected *R. lagotis* with 5 µM PMA resulted in a 212% increase in H_2_O_2_ production after 60 min in contrast to an 80% increase in the absence of PMA; thus at this time point PMA stimulated H_2_O_2_ output approximately 2.6-fold when compared to controls (p<0.001; Figure 9). Next, the ability of PKC (GF109203X; 5 µM) and MEK (U0126; 5 µM) inhibitors to affect haemocyte H_2_O_2_ production was tested. GF109203X substantially attenuated H_2_O_2_ release by PMA-stimulated haemocytes when compared to haemocytes treated with DMSO (vehicle) and PMA at all time points (p<0.01, p<0.001; Figure 9), reducing H_2_O_2_ output to levels similar to those seen under basal conditions. In addition, DMSO did not significantly affect PMA-stimulated H_2_O_2_ production when compared to that of cells treated with PMA only. U0126 also significantly reduced PMA-stimulated H_2_O_2_ production by *R. lagotis* haemocytes (Figure 10). After 60 min, the increase in H_2_O_2_ production as a result of PMA exposure was reduced by 37% (p<0.001; Figure 10).

## Discussion

### Histological observation of *T. regenti* in *R. lagotis*

We evaluated by histology haemocyte migratory/encapsulation responses triggered in *R. lagotis* by the bird schistosome, *T. regenti*. The timing of this haemocyte response should be interpreted with a tolerance of +5 h, because the snails were exposed to *T. regenti* miracidia for 5 h, after which the specimens were fixed at different time points between 1 and 92 h p.e. Taking this into account, between 1 and 6 h p.e. the response varied where some larvae were encapsulated by haemocytes while others appeared without haemocytic infiltration. In the case of *Biomphalaria alexandrina* infected with *S. mansoni*, haemocytes were also not observed around some miracidia while others underwent encapsulation 6 h p.e. [28]. Haemocytes then surrounded developing *T. regenti* sporocysts in our study between 7 and 21 h p.e., and their occurrence started to fluctuate at the latter time points. This encapsulation, however, did not lead to killing of the parasites that appeared to be morphologically intact. Similarly, compatible *S. mansoni* larvae have also been seen encapsulated but not destroyed by *B. glabrata* haemocytes in in vitro experiments or by *Biomphalaria tenagophila* fibrous cells observed in vivo [29]–[31]. Haemocytes of *R. lagotis* might be attracted towards *T. regenti* by ciliary plates shed during miracidium-mother sporocyst transformation [21]. A role of *T. szidati* ciliary plates in activating *L. stagnalis* haemocytes has been previously suggested [32], [33]. Furthermore, within haemocytes we observed microtubular aggregates that likely corresponded to the remnants of phagocytosed ciliary plates. Ciliary plates of *S. mansoni* miracidia are also phagocytosed by *B. glabrata* haemocytes [34].

Then, up until 41 h p.e., the haemocytic response against the developing *T. regenti* appeared to decline and no haemocytes were observed in the proximity of larvae between 44 and 97 h p.e. Haemocyte motility might be affected by parasite-derived components such as ESPs, which in the case of *E. paraensei* repel *B. glabrata* haemocytes [35]. Based on our observations, we suggest that the developing sporocysts of *T. regenti* escaped the cellular defence response of *R. lagotis* enabling successful parasite development. However, it is also possible that not all larvae that penetrated the snails were observed and some of these might have been destroyed after encapsulation. Both normally developing and encapsulated sporocysts of *S. mansoni* within *B. glabrata* have previously been observed [36].

Interestingly, in our laboratory-reared *T. regenti*, approximately 90% of *R. lagotis* snails become infected with the parasite while the remainder appear resistant (data not shown). This phenomenon, reduced compatibility, may be a consequence of long-term passage of the parasite in laboratory conditions [37]. However, reduced compatibility may already arise earlier as shown for the first generation of offspring of *B. alexandrina* snails susceptible to *S. mansoni* [38].

### Quantities of circulating haemocytes from uninfected and *T. regenti*-infected *R. lagotis*

Haemocyte numbers/ml haemolymph, phagocytic activity and H_2_O_2_ production were compared between uninfected and infected *R. lagotis* snails, with infected snails studied during the patent period of infection by *T. regenti*. Correlation analysis revealed that the levels of circulating haemocytes were not influenced by age (shell height) of individual *R. lagotis* from both groups. In contrast, older specimens of *Lymnaea acuminata* f. *rufescens*, *Indoplanorbis exustus* and *Ruditapes decussatus* have been shown to possess significantly higher haemocyte counts per volume of haemolymph than the younger individuals [39], [40].

Despite variation in haemocyte concentration in uninfected and infected *R. lagotis*, the infected snails had significantly more (1.8-fold) circulating haemocytes/ml haemolymph, when compared to their uninfected counterparts. Similar differences in haemocyte number were previously found between uninfected and *H. elongata*-infected *L. littorea* [15]. Infection of *B. glabrata* with *E. liei* or *E. paraensei* also results in increased numbers of haemocytes in the circulation [41], [42]. On the other hand, haemocyte concentrations appeared constant in *L. stagnalis* snails infected with *Diplostomum spathaceum* [43], suggesting that increased haemocyte number is not a general response of snails to trematode infection.

### Comparison of defence activities of haemocytes from uninfected and *T. regenti*-infected *R. lagotis* and the influence of PKC and ERK activities

In-vitro experiments with haemocytes from either uninfected or *T. regenti*-infected *R. lagotis* were particularly challenging because preparation of cell monolayers from haemolymph pools maintained on ice as done for *L. stagnalis* and *B. glabrata* [7], [17] was intractable for *R. lagotis*. The majority of haemocytes clumped during such manipulation and, therefore, aliquots of haemolymph expelled during head-foot retraction were transferred directly from the snails to the wells. Furthermore, variation in the numbers of circulating haemocytes in *R. lagotis* necessitated haemocyte counting in each experiment. Wells with haemolymph from infected snails usually contained almost twice the number of cells in haemolymph from uninfected snails.

Phagocytosis of *E. coli* bioparticles, evaluated using equal numbers of haemocytes (50,000), was approximately 50% lower in infected snails when compared to that of uninfected snails, although when considering haemolymph volume (200 µl) phagocytic activities were similar. Because bioparticles were used in excess and were found free in the incubation medium after exposure to haemocytes, we conclude that the phagocytic activity of haemocytes was not limited by *E. coli* bioparticles availability, but was supressed as a result of *T. regenti* infection. However, it remains to be determined whether individual haemocytes exhibited lower phagocytic activity generally or whether some populations were more affected than others. The increased concentration of haemocytes in infected snails which was 1.8-fold higher in comparison to uninfected snails likely compensated for the overall decreased phagocytic capacity.

Haemocytes obtained from *B. glabrata* or *L. stagnalis* infected with *E. paraensei* or *T. szidati*, respectively, also possess reduced phagocytic activity [13], [14], [44]. Such suppression was observed several days or weeks after exposure to parasites. Furthermore, phagocytic activity of haemocytes was reduced in haemocytes exposed to parasite-derived ESPs [45], [46]. Although specific bioactive molecules of *T. regenti* were not investigated in our study, the phagocytic capacity of *R. lagotis* haemocytes might be affected by products of daughter sporocysts or cercariae as these stages persist in snails in the patent phase of infection.

The PKC and ERK pathways have been found to be essential for efficient phagocytosis by haemocytes of *L. stagnalis*, *B. glabrata* or *Mytilus galloprovincialis* [7], [47], [48]. We therefore explored the possible regulatory role of PKC and ERK in phagocytosis by *R. lagotis* haemocytes. Inhibitors of PKC (GF109203X) and MEK (U0126) significantly blocked haemocyte phagocytic activity in a dose-dependent manner. At 1 µM and 10 µM, GF109203X decreased phagocytosis by 35% and 70% whereas U0126 by 33% and 67%, respectively. This supports the involvement of PKC and ERK in phagocytosis of *E. coli* bioparticles by *R. lagotis* haemocytes. Furthermore, levels of PKC and ERK phosphorylation (activation) were 57% and 55% lower, respectively, in haemocytes from infected snails compared to uninfected snails following adhesion. Thus, the reduced phagocytic activity of haemocytes from infected snails might be caused (at least partly) by supressed PKC and ERK activation in these cells. Because the level of total (phosphorylated and non-phosphorylated) ERK was not reduced in these cells it is possible that the expression of upstream signalling elements might be suppressed; these could include integrin which is known to activate ERK and to be important in cell adhesion [49], [50]. The expression of PKC protein was not studied in the current work since available antibodies are generally ineffective at recognizing PKCs in snail haemocytes (unpublished results).

Infected and uninfected *R. lagotis* haemocytes were further compared in their capacity to generate H_2_O_2_. Amplex red utilized in our study was previously used for monitoring H_2_O_2_ production by snail haemocytes [9], [24]. Basal and PMA-stimulated H_2_O_2_ production did not differ significantly between uninfected and infected snails when considering only the volume of haemolymph (50 µl). On the other hand, basal H_2_O_2_ production calculated per number of haemocytes (50,000) was significantly different, with haemocytes from uninfected snails producing more H_2_O_2_ as early as 20 min. Similarly, PMA-stimulated H_2_O_2_ production by haemocytes from uninfected snails increased significantly with time from 20 min, being approximately 2-fold higher after 60 min when compared to that of haemocytes from infected snails. The reduced capacity of haemocytes from infected snails to generate H_2_O_2_ might be important for *T. regenti* survival, as H_2_O_2_ was previously shown to be an important ROS involved in in-vitro killing of *S. mansoni* sporocysts [4]. In *L. littorea*, haemocytes from snails infected with *H. elongata* produce 2-fold less superoxide [15], a precursor of H_2_O_2_ [4], [51]. As with phagocytosis, it is possible that *R. lagotis* compensate for decreased H_2_O_2_ generation by haemocytes by increasing their number in the circulation. Nevertheless, whether all haemocytes or their proportion were inhibited remains unknown as well as components of *T. regenti* responsible for such alteration. In *B. glabrata*, PMA-stimulated production of H_2_O_2_ was significantly reduced when haemocytes were simultaneously exposed to PMA and ESPs of *S. mansoni* [10].

As PMA is an activator of PKC, a role of this kinase in the regulation of H_2_O_2_ production by haemocytes from uninfected *R. lagotis* snails was further investigated; participation of ERK signalling in this process using the MEK inhibitor (U0126) was also explored. Haemocytes exposed to GF109203X displayed substantially reduced PMA-stimulated H_2_O_2_ production that was similar to levels comparable with basal (unstimulated) H_2_O_2_ output. U0126 also significantly affected PMA-stimulated H_2_O_2_ output by snail haemocytes, although at less extent than GF109203X. Thus, PKC and ERK appear to play a role in regulating H_2_O_2_ production by *R. lagotis* haemocytes. PKC and ERK signalling were previously found to be crucial in regulation of H_2_O_2_ production by haemocytes of *B. glabrata* [10], [24] and *L. stagnalis* [9]. As already mentioned for haemocytes of infected snails, basal levels of PKC and ERK phosphorylation (activation) were significantly lower than in haemocytes of uninfected snails; lower H_2_O_2_ production by haemocytes from infected snails could therefore be the result of lower PKC and ERK activities in response to the parasite. Our study and a previous report suggesting that ESPs may attenuate PKC and ERK phosphorylation in snail haemocytes [17] support the notion that parasites modulate haemocyte defence pathways at the level of cell signalling [16] and possibly at multiple phases during development.

The present paper provides the first insights into the immunobiology of the snail *R. lagotis*, an important intermediate host of the nasal bird schistosome *T. regenti*. Histological study of the *R. lagotis* response against *T. regenti* showed that haemocytes are able to accumulate near the invading larvae, but they do not destroy the parasite. This enables further development of trematode larvae, leading to patent phase of *T. regenti* infection in snails. The phagocytic activity and capacity for H_2_O_2_ generation were suppressed in haemocytes of infected snails. Importantly, PKC and ERK that appear to regulate such responses in *R. lagotis* were also shown to be less active in haemocytes from infected snails. It is hypothesized that attenuation of both responses in haemocytes is partially compensated by increased concentration of haemocytes in the circulation of infected snails, enabling the snail to fend off other pathogens such as bacteria. Further research is needed to understand how this impacts survival and continued cercarial production of *T. regenti* in *R. lagotis*, and to determine the parasite-derived molecules responsible for alterations in *R. lagotis* haemocyte responses.
